# Electrohydraulic lithotripsy for ball valve syndrome due to stent-stone complex after endoscopic ultrasound-guided hepaticogastrostomy

**DOI:** 10.1055/a-2092-4224

**Published:** 2023-06-15

**Authors:** Moegi Mochizuki, Kenji Nakamura, Kyoko Arahata, Sakiko Takarabe, Hiroshi Kishikawa, Tadakazu Hisamatsu, Jiro Nishida

**Affiliations:** 1 Department of Gastroenterology, Tokyo Dental College, Ichikawa General Hospital, Chiba, Japan; 2 Department of Gastroenterology and Hepatology, Kyorin University School of Medicine, Tokyo, Japan

Endoscopic ultrasound-guided hepaticogastrostomy (EUS-HGS) is the standard endoscopic procedure for malignant biliary obstruction 1 2 . However, the rate of EUS-HGS-associated adverse events remains high, and various severe complications, including risk factor and rescue techniques, have been reported 3 4 5 . We present a rare case of electrohydraulic lithotripsy (EHL) for ball valve syndrome due to a stent-stone complex after EUS-HGS.

An 88-year-old woman, who underwent EUS-HGS for recurrent biliary obstruction caused by refractory transpapillary drainage due to biliary cancer, presented with acute-onset vomiting. Contrast-enhanced computed tomography revealed a calculus that had formed at the end of the stent on the gastric side. The stent was pulled into the duodenum, with the calculus visible as the leading part of the stent ( Fig. 1 ). On upper endoscopy, the EUS-HGS stent was pulled into the duodenum beyond the pyloric ring, grasped, and pulled back into the stomach ( Video 1 ). A 5-cm stent-stone complex was noted at the end of the EUS-HGS stent ( Fig. 2 ). Gastric ulceration due to compression of the EUS-HGS stent was observed ( Fig. 3 ). Since grasping forceps, an endoscopic snare, and argon plasma coagulation (APC) failed to crush the stent-stone complex, electrohydraulic lithotripsy (EHL) with 1200 shots was used. One-quarter of the complex was crushed via EHL, and the stent was trimmed with APC. However, the stent-stone complex was still unable to pass through the pharyngoesophageal junction. This was additionally crushed using EHL with 3000 shots ( Fig. 4 ). After crushing the stent-stone complex, the trimmed stent and the fragments were successfully removed ( Fig. 5 ).

**Fig. 1:**
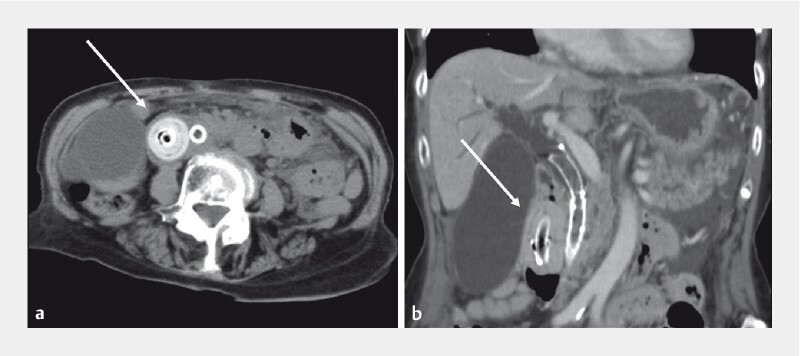
Contrast-enhanced computed tomography scans showing the calcified stent-stone complex (white arrows) formed at the end of the stent on the stomach side and pulled into the duodenum. **a** Axial view. **b** Coronal view.

**Video 1** Electrohydraulic lithotripsy for ball valve syndrome due to a stent-stone complex after endoscopic ultrasound-guided hepaticogastrostomy.


**Fig. 2:**
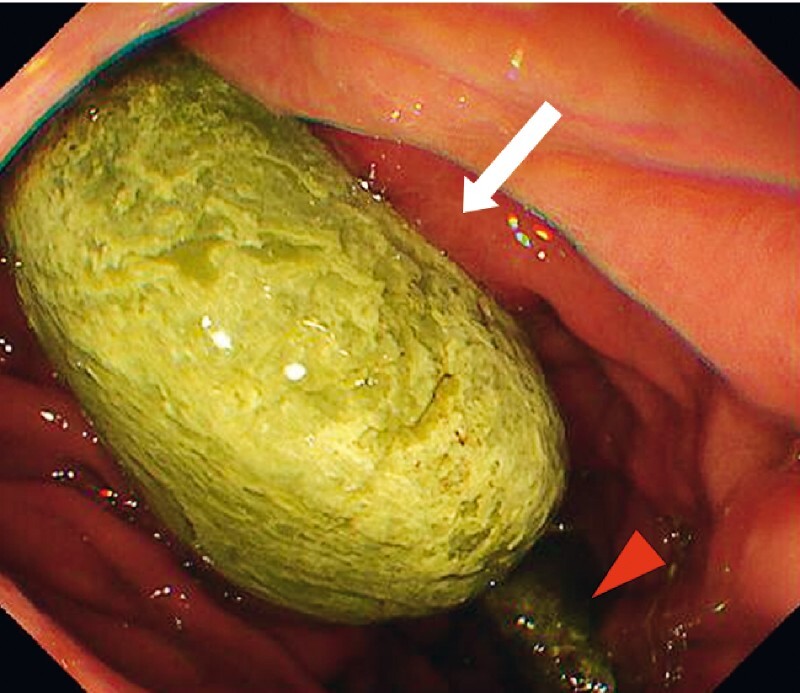
Endoscopic view of the esophagogastroduodenoscopy showing the stent-stone complex (white arrow), just after being pulled back into the stomach, formed on an endoscopic ultrasound-guided hepaticogastrostomy stent (red arrowhead) pulled into the duodenum.

**Fig. 3:**
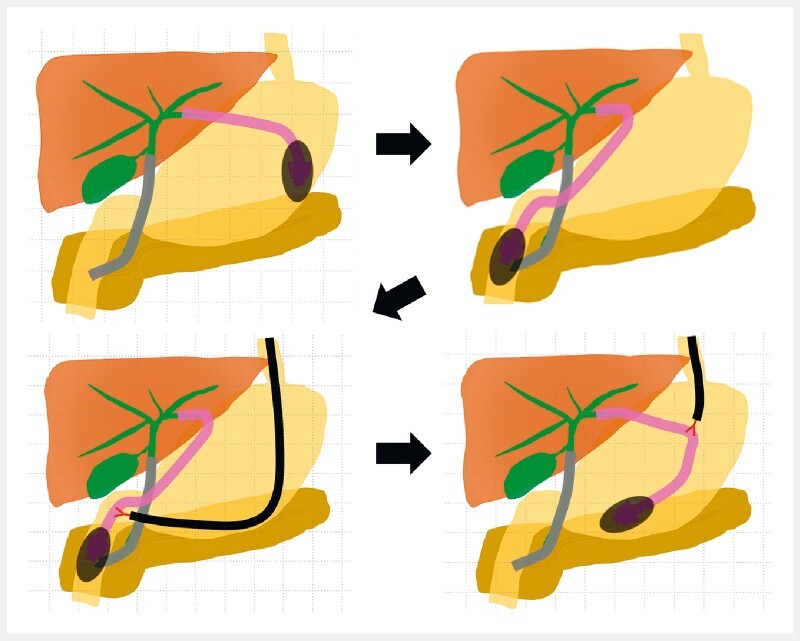
The schema of the ball valve syndrome due to a stent-stone complex formed on the endoscopic ultrasound-guided hepaticogastrostomy stent and pulled back from the duodenum into the stomach using an upper endoscopy with a grasping forceps.

**Fig. 4:**
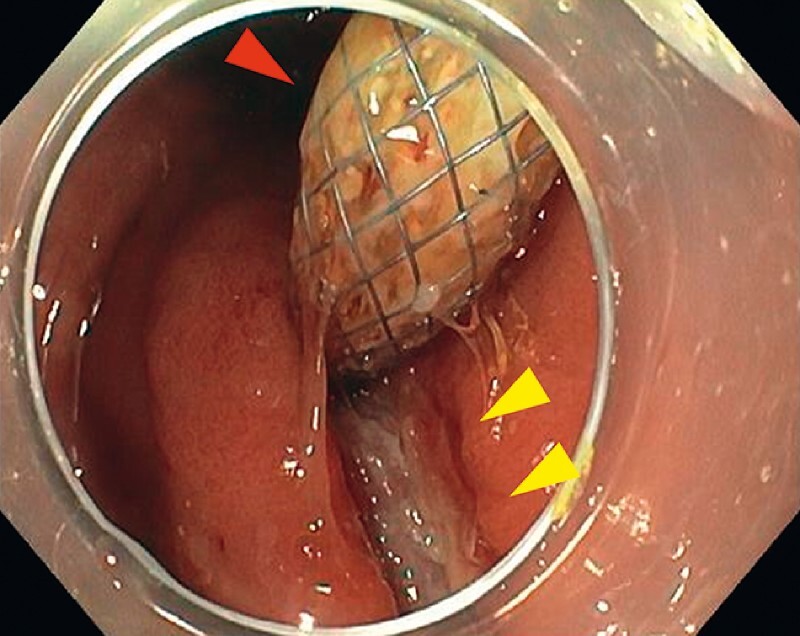
Endoscopic view of the esophagogastroduodenoscopy showing the longitudinal and deep gastric ulceration (yellow arrowheads) due to compression of endoscopic ultrasound-guided hepaticogastrostomy stent (red arrowhead) pulled into the duodenum.

**Fig. 5:**
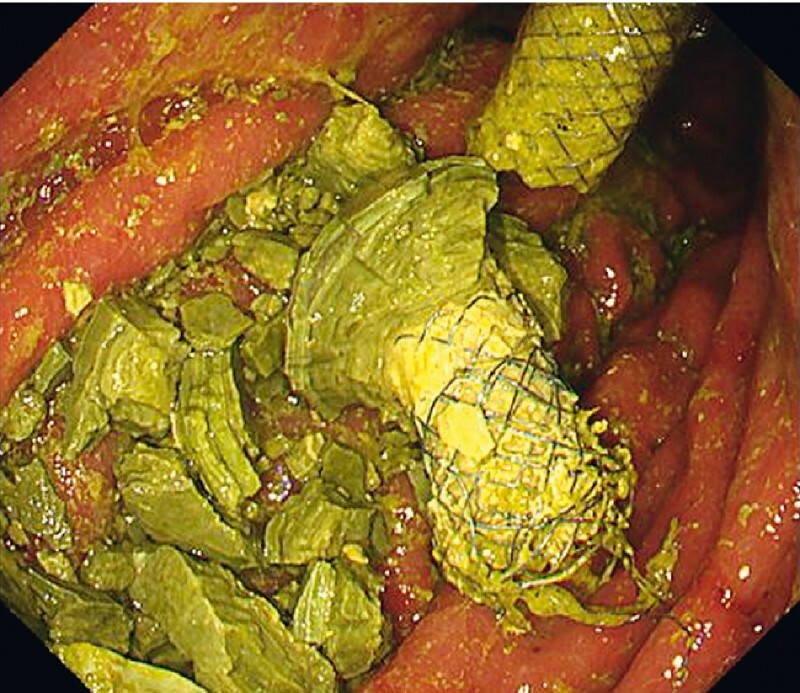
Endoscopic view of the esophagogastroduodenoscopy showing the trimmed stent end of endoscopic ultrasound-guided hepaticogastrostomy and crushed fragments of the stent-stone complex.

There are no reports of ball valve syndrome caused by a stent-stone complex formed on an EUS-HGS stent, which is a rare complication of EUS-HGS. EUS-HGS is a relatively new procedure that may cause rare complications. Clinicians should be aware of these complications and be ready to manage them using minimally invasive treatments.

Endoscopy_UCTN_Code_CCL_1AZ_2AD

## Notes

Endoscopy E-Videos

https://eref.thieme.de/e-videos
*E-Videos* is an open access online section of the journal *Endoscopy* , reporting on interesting cases and new techniques in gastroenterological endoscopy. All papers include a high-quality video and are published with a Creative Commons CC-BY license. Endoscopy E-Videos qualify for HINARI discounts and waivers and eligibility is automatically checked during the submission process. We grant 100% waivers to articles whose corresponding authors are based in Group A countries and 50% waivers to those who are based in Group B countries as classified by Research4Life (see: https://www.research4life.org/access/eligibility/ ). This section has its own submission website at https://mc.manuscriptcentral.com/e-videos
